# Metformin and bladder cancer: Drug repurposing as a potential tool for novel therapy: A review

**DOI:** 10.1097/MD.0000000000031635

**Published:** 2022-11-11

**Authors:** Yunzhu Feng, Benzhong Jia, Zhiyong Shen

**Affiliations:** a School of Clinical Medicine, Guizhou Medical University, Guiyang City, Guizhou Province, China; b Department of Urology, Affiliated Hospital of Guizhou Medical University, Guiyang City, Guizhou Province, China; c Department of Urology, Affiliated Cancer Hospital of Guizhou Medical University, Guiyang City, Guizhou Province, China.

**Keywords:** apoptosis, bladder cancer, cell cycle, metformin, signaling pathway

## Abstract

Bladder cancer (BC) is a common type of cancer worldwide. Currently, the gold standard treatment is transurethral resection of bladder tumor (TUR-Bt) accompanied by intravesical Bacillus Calmette–Guérin (BCG) instillation for patients with middle-to-high-risk non-muscle-invasive bladder cancer (NMIBC). However, intravesical BCG therapy fails in almost 50% of high risk cases, leading to NMIBC persistence or early recurrence. In these patients, the gold standard remains radical cystectomy; however, it can seriously affect the patients’ quality of life. Moreover, for patients with muscle-invasive bladder cancer (MIBC), the 5-year survival rate after radical cystectomy with neoadjuvant chemotherapy remains low. Recent discoveries have paved the way for a new era in BC treatment. Metformin is the most widely used oral hypoglycemic drug in clinical practice, being mostly used in the treatment of type 2 diabetes. Epidemiological studies have demonstrated that metformin exerts a potentially positive effect on reducing the incidence and mortality of cancer; therefore, a increasing number of studies have investigated the potential anticancer effects of metformin and its mechanisms of action. This review aims to summarize the evidence for the role of metformin in bladder cancer therapy, including how metformin mediates bladder cancer cell apoptosis.

## 1. Introduction

### 1.1. Clinical features and treatment status of bladder cancer

Bladder cancer (BC) is among the top 10 most common cancers worldwide, which caused approximately 573,000 new cases and 213,000 deaths in 2020. The incidence of BC in men is higher than that in women, and is the ninth leading cause of cancer death and the sixth most diagnosed cancer.^[1]^ BC risk factors include geography, age, sex, and exposure to carcinogens, of which smoking is the most prevalent.^[2]^ It has been estimated that half of all BC cases are caused by smoking,^[3]^ and the degree of harm corresponds to the intensity and duration of smoking.^[4]^

The most typical symptom of BC is microscopic or gross hematuria, which is clinically characterized by progression, metastasis, recurrence, and drug resistance.^[5]^ Pathologically, BC is classified into non-muscle-invasive bladder cancer (NMIBC) and muscle-invasive bladder cancer (MIBC). Clinically, approximately 70% of patients with BC are initially diagnosed with NMIBC,^[6]^ which is a localized disease that is mucosally-confined (Ta or carcinoma in situ [CIS], based on the American Joint Cancer Committee [AJCC]/Tumor-Node-Metastasis [TNM] BC staging) or invades the lamina propria (T1). In the remaining 30% of patients, BC invades deep into the bladder wall (MIBC) or metastasizes, resulting in poorer prognosis.^[7]^

International guidelines suggest that the clinicopathology of NMIBC can divide patients into low, middle, and high risk groups.^[7,8]^ The main treatment for patients with low-risk NMIBC (primary, low-grade Ta or T1 tumors, no carcinoma in situ) is transurethral resection of bladder tumor (TUR-Bt). However, for patients with middle-to-high-risk NMIBC (high grade Ta or T1, carcinoma in situ), the postoperative recurrence rate of TUR-Bt is very high because of its piecemeal technology, which could lead to tumor cells implanting into a healthy bladder lining.^[9,10]^ Studies have shown that the 5-year recurrence rate after TUR-Bt surgery is as high as 60% to 70%; however, approximately a quarter of patients will suffer from progression to invasive BC after 1 or more relapses.^[11]^ Currently, to prevent BC progression or recurrence, the main treatment plan is TUR-Bt combined with bladder infusion of immunosuppressants or chemotherapy drugs.^[12]^ Commonly used bladder infusion drugs include Bacillus Calmette–Guerin (BCG), mitomycin, adriamycin, epirubicin, and pirirubicin, among which BCG is considered the preferred bladder infusion drug and is used to prevent progression in patients with middle-to-high-risk NMIBC after surgery. Currently, TUR-Bt followed by intravesical BCG injection every 6 weeks, followed by maintenance is the gold standard treatment.^[8]^

It has been reported that the pathophysiological process of BCG perfusion might be related to increased cytokine expression in the immune response induced by drugs perfusion, which is also increased by the aggregation of monocytes and granulocytes, thus preventing tumor recurrence and progression.^[13]^ However, in about half of the patients, high-risk NMIBC persisted if intravesical BCG therapy failed, or if tumors recurred after a complete response (CR).^[14]^ In such cases, the preferred option according to the guidelines is early radical cystectomy (RC) and urinary flow diversion,^[8,15]^ which can provide good disease control in the long term; however, this treatment can negatively affect a patient’s quality of life.^[16,17]^ Until recently, non-surgical alternatives were ineffective and could only delay BC rather without achieving long-term disease control. Varubicin, an anthracycline, injected intravesically, is the only US Food and Drug Administration (FDA)-approved drug therapy, with a 21% CR rate; however, the CR is mainly temporary.^[18]^

For MIBC, the gold standard treatment is radical cystectomy (RC) combined with dissection of pelvic lymph nodes^[19]^; however, postoperative metastasis is still possible in up to 50% of patients,^[20,21]^ and half of patients die from BC within 5 years. Neoadjuvant chemotherapy (NAC) based on cisplatin could prolong the overall survival of patients undergoing MIBC radical cystectomy and has become a routine part of care.^[22]^ Although most patients with BC are sensitive to chemotherapy regimens containing cisplatin, its efficacy does not last long, with only a 20% to 40% 5-year survival rate.^[23]^

This review aims to describe the inhibitory activities of metformin against BC and its mechanism of inducing apoptosis in BC cells.

### 1.2. The hypoglycemic mechanism of metformin

In clinical practice, metformin is the most widely used oral hypoglycemic drug for the treatment of type 2 diabetes,^[24]^ which has characteristics of good efficacy and safety evidence, health economic benefit evidence, and clear clinical evidence for the prevention of cardiovascular complications as a single drug or in combination therapy.^[25]^ Metformin could significantly reduce insulin resistance and fasting plasma insulin levels. Therefore, it is considered an insulin sensitizer that appears to be associated with its beneficial effects on tyrosine kinase activity and insulin receptor expression.^[26]^ Studies have demonstrated that compared with healthy people, patients with diabetes are at a higher risk of developing cancer, partly because of increased circulating levels of growth factors such as insulin or insulin growth factor 1 and 2 (IGF-1 and 2).^[27]^

Clinical investigations have provided considerable evidence that metformin’s primary role is the reduction of glucose production in the liver, primarily via slight immunomodulation of gluconeogenesis and temporarily inhibition of the mitochondrial respiratory chain complex 1.^[28–30]^ AMP-activated protein kinase (AMPK) is activated by decreased energy in the liver, which provides the mechanism by which metformin functions in hepatic gluconeogenesis. AMPK senses intracellular energy, which participates in glucose and lipid metabolism regulation in cells.^[31]^ When the body is energy-deficient, the intracellular AMP/ATP ratio is abnormally elevated, and AMPK is activated accordingly. On the one hand, activation of AMPK can enhance the catabolic pathway of ATP production, promote glycolysis and glucose uptake, and other activities; on the other hand, AMPK inhibits the anabolic pathway that consumes ATP and reduces glycogenosis and fatty acids, cholesterol, and protein synthesis, ultimately keeping the body energy stable.^[32]^ Metformin is suggested to reduce the blood glucose level of patients with diabetes via AMPK activation in the liver and skeletal muscle cells, leading to inhibition of liver glycogen and promotion of muscle tissue glucose uptake.^[33,34]^

## 2. Anti-tumor effects of metformin

### 2.1. Metformin’s mode of action on cancer cells

Recent clinical studies in patients with type 2 diabetes have demonstrated that metformin may prevent the development of malignant tumors to a certain extent. Compared with patients with diabetes treated with other antidiabetic drugs, those treated with metformin had a reduced incidence of cancer after controlling for bias factors such as age, sex, and duration of illness.^[35]^ Bowker found that among patients with newly diagnosed type 2 diabetes followed up for 5 years, patients who took metformin to lower their blood glucose levels had significantly lower tumor-related mortality than those who took sulfonylureas or insulin.^[36]^ These results not only suggested that in patients with type 2 diabetes, metformin can reduce the incidence of malignant tumors and tumor-related mortality, but also that metformin can inhibit tumor cells to a certain degree. However, the specific mechanism by which metformin exerts these effects is still under investigation. Research has mainly focused on the effect of metformin on the tumor cell cycle and related signaling pathway activation.

#### 2.1.1. Metformin induces tumor cell cycle arrest.

The cell cycle is a highly ordered process in which 1 round of cell division progresses to the next round of division, which is the only way by which normal cells proliferate. There are 4 phases of the cell cycle: G0/G1, S, G2, and M phases. The transition of cells into the S phase from the G0/G1 phase and into the M phase from the G2 phase are the 2 important rate-limiting phases, which are regulated by cyclin–cyclin-dependent protein kinase–cyclin kinase inhibitor pathways.^[37]^ Studies on prostate cancer cells,^[38]^ kidney cancer cells,^[39]^ breast cancer cells,^[40]^ and gastric cancer cells^[41]^ have shown that metformin regulates the expression levels of G0/G1 regulatory proteins (e.g., cyclin D1, cyclin dependent kinase 4 (CDK4), and CDK6) in tumor cells, and causes G0/G1 phase cell cycle arrest. Studies on ovarian and pancreatic cancer cells revealed that cell cycle arrest at the S or G2/M phases is induced by metformin.^[41,42]^ Once the cell cycle is blocked, cell proliferation is inhibited accordingly. Thus, induction of cell cycle arrest in tumor cells might be one of the mechanisms by which metformin inhibits their proliferation.

#### 2.1.2. Anti-tumor mechanisms dependent or not on AMPK.

In cells, metformin mainly inhibits mitochondrial respiratory chain complex I, thereby interfering with intracellular ATP production^[43]^ and inducing AMPK activation.^[33]^ Activated AMPK has vital functions in cell cycle regulation, cell growth and proliferation, cell apoptosis, and autophagy.^[44–46]^ Therefore, AMPK modulators, including metformin, are regarded as important anticancer agents for the establishment of novel therapies for human cancer.^[47,48]^

After metformin induction, AMPK in cancer cells appears to be activated by hepatic kinase B1 (LKB1) on threonine 172.^[49]^ LKB1 expression is associated with cancer susceptibility. Mammalian target of rapamycin complex 1 (mTORC1) is inhibited by the LKB1/AMPK pathway, via tuberculous sclerosis complex 1 and 2 (TSC1 and TSC2) activation, which induces protein synthesis disruption and inhibition of tumor cell proliferation.^[50]^ Therefore, research on the mechanism of metformin has focused on the LKB1-AMPK-mammalian target of rapamycin (mTOR) pathway. In addition, activation of AMPK can also directly inhibit the regulatory associated protein of mTORC1 (RAPTOR), a positive regulator of mTORC1.^[51]^ MTORC1 is involved in many cellular processes; however, it primarily regulates protein synthesis, which is critical for cell growth. Clinical studies have found that mTOR activity is significantly enhanced in most patients with malignant tumors and is closely related to adverse factors, such as tumor recurrence and progression. Consequently, mTOR has become a new target in the field of tumor therapy.^[52]^

In addition, metformin inhibits mTORC1 independent of AMPK.^[53]^ For example, inhibition of the recombinant activating gene (RAG) GTPase family protein leads to mTORC1 inhibition.^[54]^ In fact, mTORC1 is recruited by recombinant activating gene (RAG) GTPases interacting with RAPTOR on the surface of the lysosome, which involves activation by Ras homolog enriched in brain (Rheb). Moreover, metformin directly inhibits the mTORC1 activator, Ragulator.^[55]^

Finally, mTORC1 inhibition activates the mechanisms that lead to cancer cells death. Phagocyte formation during autophagy is inhibited by mTORC. It also inhibits UNC-51-like autophagy activating kinase 1 (ULK1), a kinase that is necessary to induce autophagy, which is also inhibited by mTORC.^[56]^ Metformin activates AMPK, which promotes the initiation of autophagy via serine 1345 phosphorylation of TSC2, which then inhibits the mTORC1 complex.^[57,58]^ In addition, UNC-51-like autophagy activating kinase 1 (ULK1) is directly phosphorylated by AMPK, leading to mTOR-independent autophagy.^[56]^ During cancer cell apoptosis, autophagy induction enhances cysteine caspase-dependent apoptosis.^[59]^ In adipocytes, AMPK activates apoptosis via eukaryotic initiation factor 2 α (eIF2α) in response to stimulation by 5-aminoimidazole-4-carboxamide ribonucleotide formyltransferase/IMP cyclohydrolase (AICAR).^[60]^ In addition, AMPK activation stimulates p53 serine 46 phosphorylation, leading to programmed cell death via type 1 apoptosis.^[61]^

#### 2.1.3. Insulin and insulin-like growth factor-1 (IGF-1)-dependent anti-tumor mechanisms.

Insulin and IGF-1 are growth factors that stimulate cell survival and mitosis, respectively. When IGF-1 binds to its receptor, IGF-1R, phosphatidyl inositol 3-kinase (PI3K) is activated, followed by PI3K activation of protein kinase B (AKT) to form the IGF-1/PI3K/AKT pathway.^[62]^ Moreover, the insulin receptor (IR) can transmit signals to Ras/Raf/extracellular regulated kinase (ERK) via growth factor receptor-bound protein 2 (GRB2) to form the IR/GRB2/Ras/Raf/ERK pathway. Both pathways play crucial roles in regulating cell survival and proliferation. Insulin and IGF-1 receptors are highly expressed in breast, liver, colon, pancreatic and skin cancers. Metformin can stop the feedback regulation of the IGF-1R signaling pathway and synergistically kill tumor cells when combined with IGF-1R inhibitors.^[63]^

### 2.2. Effects of metformin on tumor microenvironment

The tumor microenvironment (TME) is the internal environment where tumor cells generate and exist, which is characterized by hypoxia, chronic inflammation, and immunosuppression. The TME plays a vital role in the occurrence, development, and drug resistance of tumors. Metformin plays an anti-tumor role by ameliorating the hypoxia, chronic inflammation, and immunosuppression in the TME.

#### 2.2.1. Regulation of tumor angiogenesis.

The tumor vascular structure is the result of multiple angiogenic signals from the tumor cells and TME components. Metformin directly inhibits angiogenesis by inhibiting endothelial cells, and analysis of angiogenesis in mice with matrix glue embolization demonstrated that the effect of metformin treatment was correlated with reduced angiogenesis,^[64]^ possibly related to AMPK activation and subsequent downregulation of the ERK pathway. Metformin indirectly affects angiogenesis by inhibiting the angiogenesis signaling pathway. Metformin-activated AMPK/mTOR signal transduction pathway can reduce the expression and stability of hypoxia-inducible factor-1α (HIF-1α) in tumor cells. Decreased expression of HIF-1α targeted genes, including the encoding vascular endothelial growth factor, leads to the reduction of tumor vessel size and vascular density, thus playing an anti-tumor role.^[65,66]^

#### 2.2.2. Regulation of inflammatory factors.

The inflammatory response is closely associated with tumors, and patients with tumors have high levels of inflammatory markers.^[67,68]^ Metformin can reduce the levels of tumor necrosis factor-α (TNF-α), interleukin-6 (IL-6), macrophage migration inhibitor, C-reactive protein, and other inflammatory marker in the human body, thus exerting an anti-tumor role.

#### 2.2.3. Immune activation.

A study found that metformin can improve the metabolic pattern in the TME, because of the effects of metformin on tumor cell metabolism, leading to a lack of oxygen in the TME, which in turn increases the number of tumor-infiltrating lymphocytes and rescues T cells from the effects of anoxia, thereby restoring and enhancing the capacity of killer T cells.^[69]^

## 3. Metformin and bladder cancer

Metformin-induced cancer cell death occurs through various mechanisms. Metformin can inhibit BC by inducing apoptosis or cell cycle arrest, by activating various signaling pathways, blocking BC stem cell proliferation, and enhancing the efficacy of targeted therapy and chemotherapy drugs in BC cells.

### 3.1. Cell apoptosis and cell cycle arrest induction

Research indicates that the cell cycle in tumor cells can be stagnated by metformin, thus showing anti-tumor activity. Metformin inhibits cyclin D1 expression, which causes G0/G1 phase cell cycle inhibition in BC 5637 and T24 cells, thereby significantly reducing the proliferation of BC cells.^[70]^

Apoptosis is the spontaneous and orderly death of cells under the regulation of genes, which maintains the stability of the internal environment. Metformin can significantly enhance BC cell sensitivity to TNF-related apoptosis-inducing ligand (TRAIL) by inhibiting the mTOR/ribosomal protein S6 kinase B1 (S6K1) signaling pathway and then downregulating the expression of the cell-type FAS-associated death domain (FADD)-like interleukin-1β-converting enzyme (FLICE) inhibitory protein (c-FLIP), which significantly increases the apoptosis of BC cells.^[71]^

### 3.2. Metformin-induced activation of various signaling pathways

#### 3.2.1. The AMPK/mTOR signaling pathway.

Metformin directly inhibits the growth of insulin-induced malignant tumors in an AMPK/mTOR dependent manner and can affect tumorigenesis by regulating IGF.^[72]^ Metformin reduces the binding of ligand IGF to the receptor IGF-R through the AMPK/mTOR pathway and indirectly downregulates the insulin signaling pathway in tumors, thereby inhibiting tumor growth.^[55]^

#### 3.2.2. The signal transduction and activator of transcription-3 (STAT3) signaling pathway.

In vitro experiments have shown that metformin can effectively inhibit STAT3 activation via a mechanism related to cell cycle inhibition, decreased cell invasion, migration, and proliferation and increased apoptosis of BC cells. These findings provide evidence that metformin blocks precancerous lesion progression to an invasive tumor by downregulating STAT3 pathway activation, which is very significant in preventing NMIBC progression to MIBC.^[73]^

#### 3.2.3. The cellular FLICE inhibitory protein (Long) (c-FLIPL)/trail/procaspase signaling pathway.

C-FLIP_L_ is a key substance that affects the sensitivity of BC to TRAIL, which can competitively bind FAS-associated protein with the death domain (FADD) to inhibit the self-shearing of procaspase-8, thus inhibiting apoptosis.^[74]^ Metformin can inhibit the phosphorylation of mTOR/S6K1, thereby inhibiting c-FLIP_L_ translation, downregulating its expression, and counteracting its inhibitory effect on death-inducing signaling complex (DISC)-induced apoptosis.^[75]^

### 3.3. Metformin’s effects on BC stem cells

The infinite proliferation of tumor stem cells provides sufficient cell numbers for tumor growth and recurrence, whereas the movement and migration of tumor stem cells provide the necessary preconditions for tumor metastasis and invasion. Research has revealed that metformin downregulates prostaglandin E2 (PGE2) by inhibiting the cyclooxygenase-2 (COX2) signaling pathway. PGE2 combined with STAT3 inhibits BC stem cell proliferation.^[76]^

### 3.4. Metformin combined with targeted therapy or chemotherapy to inhibit bladder cancer

Cancer cells might differentiate into different subpopulations during proliferation, which could lead to differences in the cell growth rate, growth cycle, and drug sensitivity among subpopulations, thereby limiting the efficacy of single drug use. Drug combinations can exert synergistic effects, not only reducing the toxicity and side effects of drugs, but also reducing the resistance to single drugs, thus exerting a better anticancer effect. The following is a summary of the mechanisms by which metformin combined with different drugs enhances the anti-bladder tumor effect.

#### 3.4.1. Metformin combined with gefitinib.

3-(4,5-dimethylthiazol-2-yl)-2,5-diphenyltetrazolium bromide (MTT) assays of MB49, T24, and UMUC3 BC cell lines showed that the inhibition rate of gefitinib combined with metformin was significantly higher than that of either drug alone. Further analysis showed that the gefitinib-metformin combination synergistically inhibited BC growth through the epidermal growth factor receptor (EGFR), AKT, and ERK-linked AMPK pathways.^[70]^ Therefore, the combination of these 2 drugs strongly inhibits cancer cell proliferation, clonal colony formation, and induces cancer cell apoptosis. This drug combination has great therapeutic and market potential for BC treatment.

#### 3.4.2. Metformin combined with theprubicin (THP).

Pirubicin has similar anti-tumor effects that are similar to those of doxorubicin in inhibiting DNA synthesis. Therefore, pirubicin has become the first-line drug for local bladder infusion chemotherapy after tumor resection. THP inhibits BC cell proliferation by activating AMPK and inhibiting tumor RNA synthesis. In vitro experiments showed that the THP-metformin combination could significantly reduced BC cell proliferation compared to THP alone. Metformin significantly enhanced the killing activity of pirubicin against T24 cells via a mechanism related to metformin-induced inhibition of X-linked inhibitor of apoptosis protein (XIAP). XIAP expression and its interaction with caspase-9, caspase-7, and caspase-3 in T24 cells promoted the effect of pirubicin on caspase-9, caspase-7, and caspase-3 in T24 cells. Activation of caspase-3 is associated with apoptosis.^[77]^

#### 3.4.3. Metformin combined with cisplatin.

Metformin combined with cisplatin inhibits tumor cell angiogenesis, growth, proliferation, and survival via the mTOR/AKT pathway. In combination with TRAIL, metformin activates caspases, leading to apoptosis. Compared with metformin and cisplatin alone, the cisplatin-metformin combination can enhance the anti-tumor effect on BC in vivo and in vitro.^[78]^

In summary, metformin combined with a variety of different targeted therapies or chemotherapy agents exerts anti-BC effects through different molecular mechanisms. When combined with gefitinib, metformin can activate the AMPK and epidermal growth factor receptor (EGFR) pathways to synergistically inhibit the growth of BC; when combined with pirubicin, AMPK activation is enhanced, and RNA synthesis is inhibited, which increases tumor suppression. When combined with cisplatin, metformin increased the AKT/mTOR pathway-mediated inhibition of cancer.

### 3.5. Metformin combined with natural agents or other drugs

Metformin alters metabolism from anabolic to catabolic metabolism via its effects on mTORC1 and AMPK. Considering the functions of mTORC1 and AMPK in physiology, pathophysiology, and pathology, and the acknowledged safety of metformin or its combinations and their proposed therapeutic roles, targeting AMPK, mTORC1, or other metabolic regulatory pathways is a valid option.^[79]^

The effect of metformin combined with natural agents or other drugs on bladder cancer cells has been extensively studied. The antineoplastic effects of metformin are evident at lower concentrations when administered with other agents such as mTOR inhibitors. Interestingly, the mTOR inhibitor rapamycin has anti-tumor activity and induces a BCG-mediated immune response.^[79]^

## 4. Conclusions and further perspectives

At present, the therapeutic effect of BC is limited due to the high postoperative recurrence rate and the limited efficacy of immunosuppressive agents or chemotherapy drugs. Therefore, novel cellular targets and new molecular therapies are urgently required. Various clinical studies and in vitro cell experiments indicate that metformin appears to be a good candidate for the development of new therapies for BC. Metformin, as a beneficial metabolic drug with multiple molecular targets for diabetes, not only plays an important therapeutic role in regulating metabolic homeostasis, but also exerts an anti-BC effect through inducing apoptosis or cell cycle arrest via activating a variety of signaling pathways, blocking the proliferation of BC stem cells, and enhancing the efficacy of targeted therapy and chemotherapy drugs on BC cells. However, further studies are needed to confirm the safe dose range of metformin in patients with bladder cancer, and whether metformin can effectively inhibit the recurrence of bladder cancer.

## Author contributions

**Conceptualization:** Yunzhu Feng, Benzhong Jia, Zhiyong Shen.

**Formal analysis:** Yunzhu Feng, Benzhong Jia, Zhiyong Shen.

**Funding acquisition:** Yunzhu Feng, Benzhong Jia.

**Supervision:** Benzhong Jia, Zhiyong Shen.

**Writing – original draft:** Yunzhu Feng.

**Writing – review & editing:** Yunzhu Feng, Benzhong Jia, Zhiyong Shen.
